# Complete Genome Sequences of Oryctes rhinoceros *nudivirus* Strains Detected in Haplotype-G Oryctes rhinoceros from Johor, Malaysia

**DOI:** 10.1128/mra.00019-23

**Published:** 2023-02-28

**Authors:** Erise Anggraini, Ganesan Vadamalai, Lih Ling Kong, Mazidah Mat, Wei Hong Lau

**Affiliations:** a Department of Plant Protection, Faculty of Agriculture, Universiti Putra Malaysia, Serdang, Selangor, Malaysia; b Department of Plant Pests and Diseases, Faculty of Agriculture, Universitas Sriwijaya, Indralaya, Ogan Ilir, South Sumatra, Indonesia; c Institute of Plantation Studies, Universiti Putra Malaysia, Serdang, Selangor, Malaysia; d Malaysian Agricultural Research and Development Institute, Persiaran MARDI-UPM, Serdang, Selangor, Malaysia; DOE Joint Genome Institute

## Abstract

Two members of the species Oryctes rhinoceros *nudivirus* (OrNV) were detected in *O. rhinoceros* haplotype G beetles collected from an oil palm plantation in Kluang and a wild coconut tree in Batu Pahat (Johor, Malaysia). OrNV strain Kluang comprised 125,794 bp, encoding 125 open reading frames (ORFs), while OrNV strain Batu Pahat comprised 124,925 bp, encoding 126 ORFs.

## ANNOUNCEMENT

Oryctes rhinoceros *nudivirus* (OrNV) (genus *Alphanudivirus*, family *Nudiviridae*) was used to control the outbreak of coconut rhinoceros beetles (CRB; *Oryctes rhinoceros*) (1, 2). Whole-genome sequences for five OrNV strains are currently available at GenBank: (i) Ma07, detected in an unknown haplotype of CRB in Malaysia (3); (ii) SI, detected in CRB haplotype G (CRB-G) in the Solomon Islands (4); (iii) LiboV, detected in an unknown CRB haplotype in Riau (5); (iv) OrNV-Palau1; and (v) OrNV-X2B, detected in CRB-G in Palauan (6). Here, we report the complete genome sequences of OrNV isolates Kluang, detected in third-instar larvae of CRB-G collected on an oil palm plantation in 2020 (global positioning system [GPS] coordinates, 2.0248117446899414, 103.25872039794922), and Batu Pahat, detected in CRB-G collected from a wild coconut tree (GPS coordinates, 1.720853, 103.053085) in 2021 in Johor, Malaysia.

Gut tissue from diseased CRB larvae showing symptoms of a swollen abdomen and prolapsed rectum was frozen in liquid nitrogen, ground with a mortar and pestle, and then subjected to DNA extraction using the NucleoBond RNA soil kit (Macherey-Nagel GmbH & Co. KG). A DNA sample of 1 μg was used as the input material for library preparation. Libraries were generated using the NEBNext Ultra II DNA library prep kit (NEB, USA), including 15 cycles of PCR amplification of the libraries. The quantity and quality of libraries were analyzed using the Agilent 2100 Bioanalyzer. The libraries were sequenced using the Illumina NovaSeq 6000 platform at Novogene Biological Information Technology Co., Ltd. (Singapore).

A total of 83,541,020 and 81,506,034 paired-end 150-bp reads were generated from the total DNA of OrNV Kluang and OrNV Batu Pahat, respectively. The quality of the raw reads was checked using FastQC v0.11.9 (7). Low-quality reads (Q ≤ 28) were removed using fastp v0.20.1 (8). The clean reads were assembled *de novo* using MetaviralSPAdes (9), which comprised three independent steps: viralAssembly, viralVerify, and viralComplete. Multiple contigs were obtained, but only one contig in each sample showed 98.6% and 97.9% genome completeness scores for OrNV Kluang and OrNV Batu Pahat, respectively, using OrNV Ma07 (GenBank accession no. NC_011588) as the reference. The protein-coding genes of each OrNV were predicted using Prokka (10). The Glimmer3 (11) plugin Geneious Prime v2023.0.3 (Biomatters Ltd., New Zealand) was used to analyze other open reading frames (ORFs). The predicted ORFs were annotated against the PfamA database v32.0 (12) using HMMER3 v3.3 with the hmmsearch function (13). All tools were run with default parameters unless otherwise specified.

**FIG 1:**
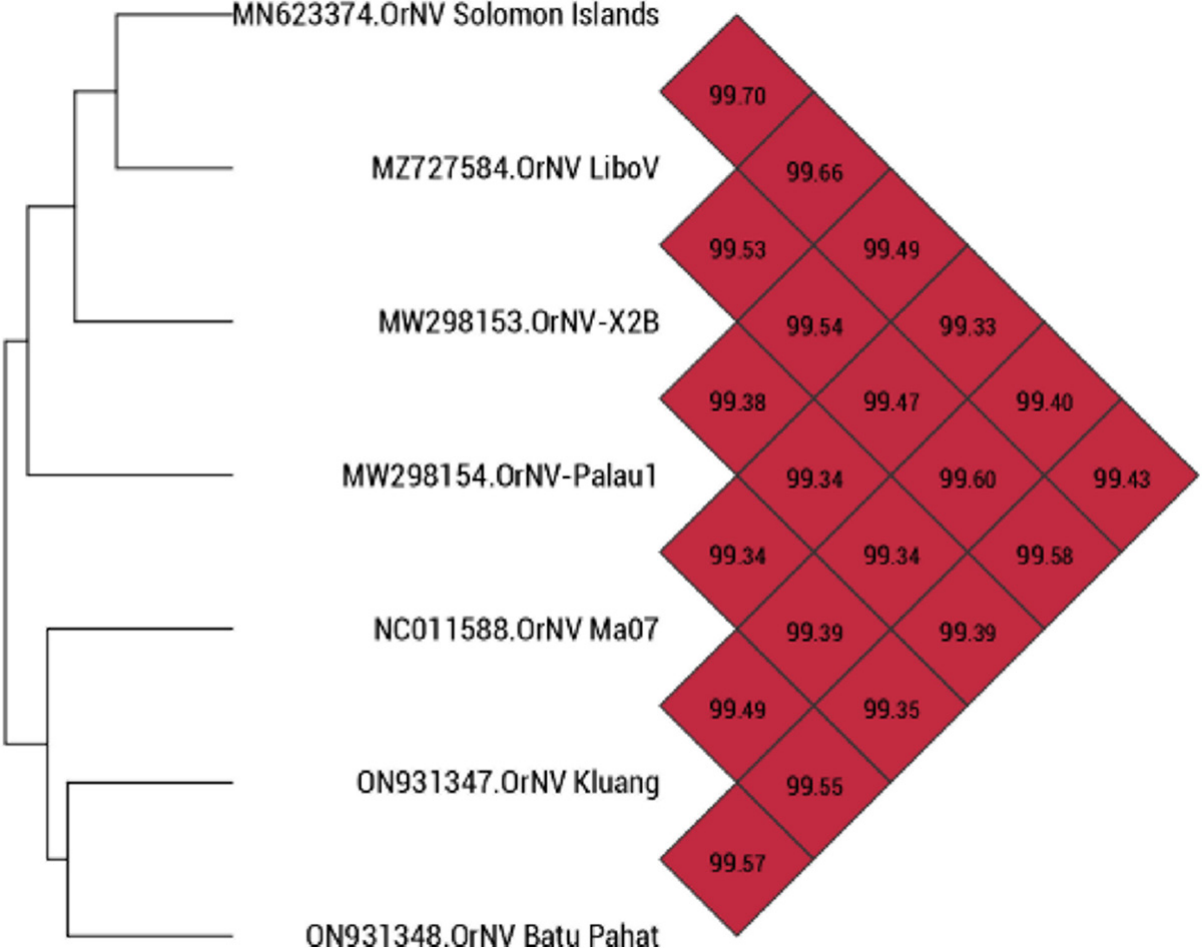
Phylogenetic tree and average nucleotide identities of OrNV genomes, calculated using OrthoANI v0.93 (14). Values in the colored matrix boxes indicate the percentage similarity among the genomes.

The genome of OrNV Kluang consisted of 125,794 bp (GC content, 41.7%), while the genome of OrNV Batu Pahat consisted of 124,925 bp (GC content, 41.7%), with coverages of 275× and 81×, respectively. The genomes of OrNV Kluang and Batu Pahat encoded 125 and 126 ORFs, respectively, and both encoded 79 ORFs associated with a predicted function. The average nucleotide identities (ANI) among the available OrNV genomes is summarized in Fig. 1. The complete genomes of OrNV presented here provide a valuable resource for future study toward biological and geographical insight into OrNV infection within or outside Malaysia.

### Data availability.

The whole-genome sequences described here have been deposited at GenBank under accession no. ON931347 and ON931348 and SRA accession no. SRR20323936 and SRR20323938.
